# Arthralgia adverse events due to immune-checkpoint inhibitors for lung cancer patients: a systematic review and meta-analysis

**DOI:** 10.3389/fonc.2023.1258287

**Published:** 2023-09-29

**Authors:** Defang Zou, Xiaoping Wang, Yamin Sun, Xi Wang, Chang Lu, Aiyun Wang, Xia Wang, Yan Yang

**Affiliations:** ^1^ Jiangsu Key Laboratory for Pharmacology and Safety Evaluation of Chinese Materia Medica, School of Pharmacy, Nanjing University of Chinese Medicine, Nanjing, China; ^2^ Jiangsu Joint International Research Laboratory of Chinese Medicine and Regenerative Medicine, Nanjing University of Chinese Medicine, Nanjing, China; ^3^ Jiangsu Collaborative Innovation Center of Traditional Chinese Medicine (TCM) Prevention and Treatment of Tumor, Nanjing University of Chinese Medicine, Nanjing, China; ^4^ Department of Orthopedics, PLA Strategic Support Force Characteristic Medical Center, Beijing, China; ^5^ Department of Anesthesiology, Third Affiliated Hospital of Naval Medical University, Shanghai, China; ^6^ Nanjing University of Chinese Medicine, Nanjing, China; ^7^ Oncology Department, the Second Affiliated Hospital of Nanjing University of Chinese Medicine (Jiangsu Provincial Second Chinese Medicine Hospital), Nanjing, China; ^8^ China Science and Technology Development Center of Chinese Medicine, Beijing, China

**Keywords:** immune-checkpoint inhibitors, programmed cell death-1, lung cancer patients, systematic review, meta-analysis

## Abstract

**Background:**

Immune agents targeting Programmed cell death-1 (PD-1) are a new type of cancer treatment drugs. By inhibiting the interaction between PD-1 and PD-L1, the ability of the immune system to attack tumor cells is enhanced. These immune preparations have shown significant efficacy in the treatment of various malignant tumors. However, like other drugs, immune preparations targeting PD-1 may also cause side effects, including arthralgia. Therefore, we conduct a meta-analysis to assess whether immune-checkpoint inhibitors targeting programmed cell death-1 in lung cancer patients will lead to arthralgia adverse events.

**Methods:**

We conducted a comprehensive search across multiple databases, including PubMed, Medline (Ovid), Web of Science, Cochrane, Embase, Scopus, CKNI, Wang fang, VIP database, Sino Med, and Clinical Trails, to identify relevant studies. The search encompassed articles published up until June 20th, 2023. The primary outcome is adverse events about arthralgia and secondary outcomes are any other related with arthralgia. Data extraction was carried out by two independent individuals, and the Cochrane Risk of Bias tool version 2.0 was employed to assess the included studies. The systematic review and meta-analysis were conducted using RevMan 5.3 software.

**Results:**

12 studies are included in the meta-analysis. All included studies were determined to have a low risk of random sequence generation bias. The meta-analysis result showed that arthralgia RR = 1.11, 95% CI [0.88, 1.40], I^2 ^= 56%, back pain RR = 1.86, 95% CI [1.07, 3.26], I^2 ^= 84%, myalgia RR = 0.49, 95% CI [0.27, 0.88], I^2 ^= 86% and muscular pain RR = 1.97, 95% CI [1.40, 2.77], I^2 ^= 23%.

**Conclusion:**

The use of targeted inhibitors may lead to an increased incidence of back pain, while potentially reducing the occurrence of myalgia. On the other hand, immune-checkpoint inhibitors targeting programmed cell death-1 in lung cancer patients may not cause arthralgia and muscular pain.

## 1 Introduction

Lung cancer is a prevalent malignant tumor, and its treatment options include surgery, radiotherapy, chemotherapy, and immunotherapy (1). Immunotherapy has emerged as a new treatment option for lung cancer by activating the patient’s immune system and enhancing their ability to attack tumors. One of the commonly used immunotherapeutic drugs is the immunocheckpoint inhibitor targeting Programmed cell death -1 (PD-1) (2). However, this drug can also cause adverse events, including arthralgia (3).

PD-1 inhibitors are immune checkpoint inhibitors that activate the immune system and enhance its ability to attack tumors by inhibiting the binding of PD-1 and its ligand PD-L1 (4). However, PD-1 inhibitors can also cause adverse events, including arthralgia. Arthralgia is a common adverse event of PD-1 inhibitor treatment, characterized by joint swelling, pain, stiffness, and other symptoms. These symptoms can affect the patient’s quality of life and even hinder the progress of treatment (5).

The mechanism of arthralgia is not entirely clear, but it may be related to the immune system activation caused by PD-1 inhibitors. PD-1 inhibitors can activate the immune system, causing immune cells to attack normal tissues and trigger inflammatory reactions. This inflammatory reaction may affect the normal function of joints, leading to arthralgia and other symptoms (6, 7).

When patients experience adverse events such as arthralgia, doctors usually take appropriate treatment measures based on the severity of symptoms and individual patient conditions (8). Nonsteroidal anti-inflammatory drugs, steroids, and other drugs can be given to alleviate symptoms. During the treatment process, doctors also need to closely monitor the changes in the patient’s condition and adjust the treatment plan in a timely manner. Nonsteroidal anti-inflammatory drugs are commonly used to relieve arthralgia (9). The drug can alleviate arthralgia and other symptoms by inhibiting inflammatory reactions. However, Nonsteroidal anti-inflammatory drugs can also cause adverse events, such as gastrointestinal bleeding, renal function damage, etc. Therefore, when using Nonsteroidal anti-inflammatory drugs, attention should be paid to the dosage and medication time to avoid unnecessary adverse events. In addition to drug treatment, patients can also take some self-management measures to alleviate arthralgia and other symptoms. For example, appropriate exercise can be performed to maintain joint flexibility and mobility; Pay attention to diet and avoid excessive intake of fat and sugar; Maintain a good mindset and avoid excessive anxiety and tension (10).

When lung cancer patients receive PD-1 inhibitor treatment, they may experience adverse events such as arthralgia, which require timely diagnosis and treatment. Doctors need to develop personalized treatment plans based on the individual situation of patients to improve treatment effectiveness and reduce the occurrence of adverse events (11). Patients also need to actively cooperate with the doctor’s treatment, take self-management measures, alleviate arthralgia and other symptoms, and improve the quality of life (12). Therefore, we conduct a systematic review and meta-analysis to assess the arthralgia adverse events due to immune-checkpoint inhibitors targeting programmed cell death-1 in lung cancer patients.

## 2 Methods

### 2.1 Database selection and search strategy

A thorough search of multiple databases, including PubMed, Medline (Ovid), Web of Science, Cochrane, Embase, Scopus, CKNI, Wang fang, VIP database, Sino Med, and Clinical Trails, was conducted to identify relevant studies. The search was conducted until June 20th, 2023. The search strategy employed for Medline (Ovid) is outlined in **Table 1**.

**Table 1:** The search strategy of Medline (Ovid).

Search order	Search strategy
#1	exp small cell lung carcinoma/
#2	exp SCLC/
#3	small cell.ab,ti.
#4	oat cell.ab,ti.
#5	lung cancer*.ab,ti.
#6	lung carcinom*.ab,ti.
#7	lung neoplasm*.ab,ti.
#8	lung tumo*.ab,ti.
#9	Or/1-8
#10	exp immunotherapy/
#11	Programmed Death‐Ligand 1 Inhibitor*.ab,ti.
#12	exp Immune Checkpoint Inhibitors/
#13	exp PD‐1 Inhibitor*/
#14	PD‐L1 Inhibitor*.ab,ti.
#15	or/10‐14
#16	exp arthralgia/
#17	exp adverse events/
#18	arthralgia.ab,ti.
#19	Or/16-18
#20	9and 15 and 19

### 2.2 Inclusion criteria

a) Randomized controlled trials (RCTs);
b) patients have adverse events with arthralgia or related symptoms;
c) Patients using immune-checkpoint inhibitors targeting programmed cell death-1 in lung cancer patients as the main treatment.

### 2.3 Exclusion criteria

a) Not report related outcomes.
b) Studies with an observational design were included in the analysis.
c) Insufficient information regarding the baseline characteristics was noted.

### 2.4 Primary outcome

Adverse events about arthralgia

### 2.5 Secondary outcomes

a) Any other related with arthralgia
b) Other symptoms related to bone pain including back pain, myalgia, muscular pain and etc.

### 2.6 Method of data extraction

Two independent reviewers (Defang Zou and Xiaoping Wang) conducted data extraction using a standardized form that included study demographics, baseline characteristics, study design, intervention methods, outcome measures, and results. In case of any discrepancies, the reviewers resolved them through discussion, and a third review author was consulted if necessary.

### 2.7 Bias risk assessment

The risk of bias in the included studies was assessed by two authors using the Cochrane Handbook for Systematic Reviews of Interventions Version 6.0 (updated July 2019) risk of bias assessment tool. Any discrepancies were resolved through consensus. The assessment tool evaluated seven items, including random sequence generation, assignment concealment, blinding of participants and personnel, blinding of outcome assessment, incomplete outcome data, selective reporting, and other bias. The items were categorized as green, yellow, and red colors and “+”, “-”, “?”, indicating “low,” “high,” and “unclear” risk of bias.

### 2.8 Publication bias assessment

To assess publication bias related to the primary outcome measures, funnel plots were generated using RevMan 5.3 software.

### 2.9 Statistical analysis

The Review Manager software (RevMan version 5.3, Cochrane Collaboration, Oxford, UK) was utilized to conduct statistical analyses. The effect size for merging the continuous variables in the study was determined using WMD and a 95% CI. For merging the binary variables, RR and a 95% CI were used as the effect size. Initially, a heterogeneity test was conducted on the included studies. Sensitivity analysis was performed to investigate any significant clinical or methodological heterogeneity. Statistical heterogeneity was assessed using I2 and P values.

### 2.10 Heterogeneity analysis

The presence of heterogeneity among trial results was evaluated using the P value and I² statistic. When more than two articles were included, heterogeneity was assessed. If the I² value exceeded 50%, the random effect model was used based on clinical heterogeneity. To identify the source of heterogeneity, subgroup analysis, sensitivity analysis, and funnel plots were employed. The statistical calculations were performed using RevMan 5.3 software.

## 3 Results

### 3.1 Literature search

**Figure 1:**
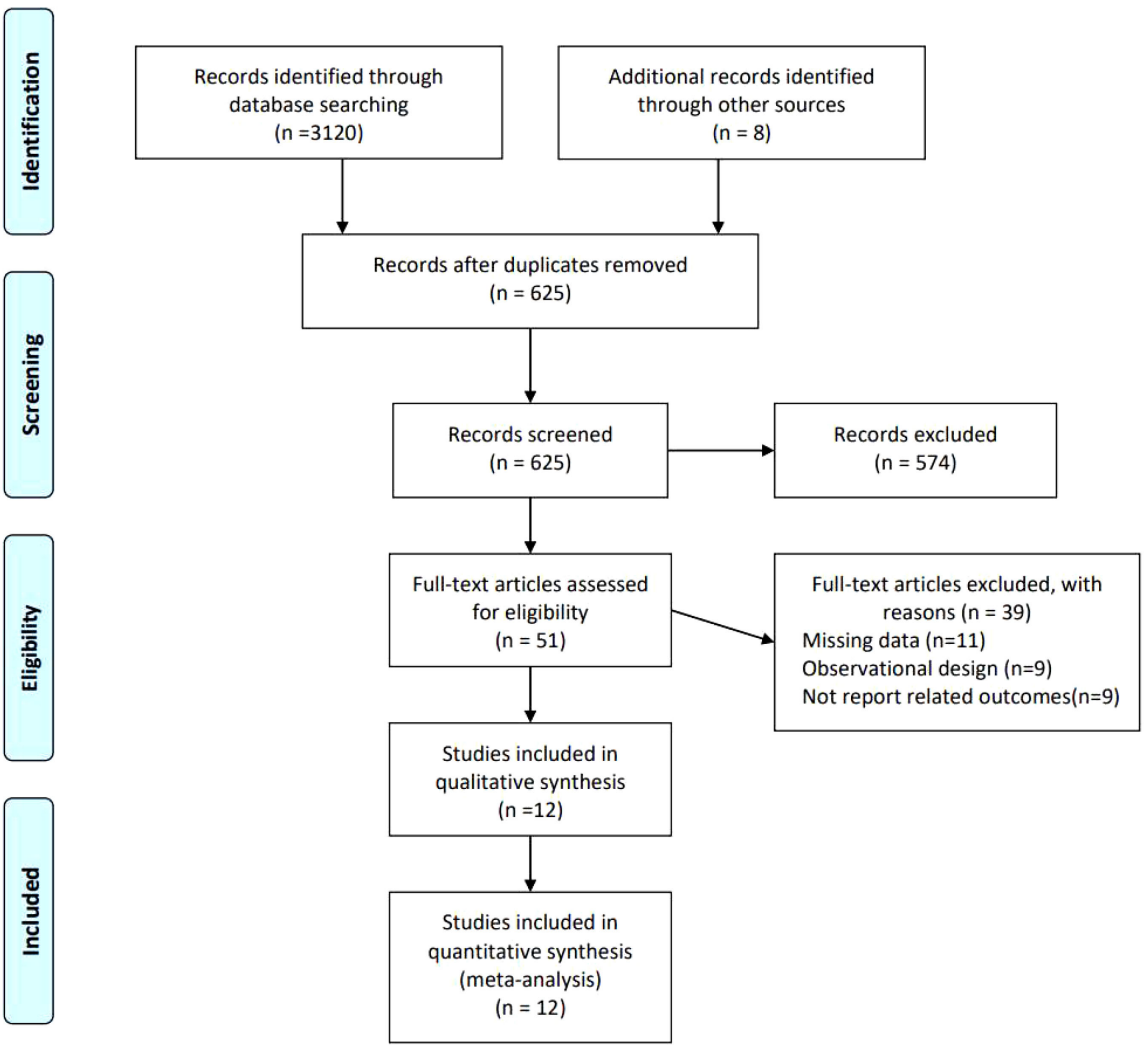
Flowchart of study selection.

We conducted a comprehensive search on 8 databases, namely PubMed, Medline (Ovid), Web of Science, Cochrane, Embase, Scopus, CKNI, Wang fang, VIP database, Sino Med, and Clinical Trials, until June 16th, 2023. A total of 3120 records were identified through the database searching process, while an additional 8 records were found through other sources. After removing duplicates, we collected 625 unique records after duplicates removed. Out of these, 51 articles were assessed for eligibility, and ultimately, 12 studies were finally included in the meta-analysis (**Figure 1**).

### 3.2 Characteristics of include studies

12 studies characteristics information are collected in **Table 2**. The difference is discussed by the third author or the whole group. The characteristics of include studies can be found in **Table 2**.

**Table 2:** Characteristics of include studies.

Study	Exp. (N)	Exp. Intervention	Con. (N)	Con. Intervention	Study Design	Outcomes	Registration Number
Brahmer2015 (13)	131	Nivolumab	129	Docetaxel	RCT	ac	NCT01642004
Borghaei2015 (14)	287	Nivolumab	268	Docetaxel	RCT	ac	NCT01673867
Herbst2016 (15)	339	Pembrolizumab	309	Docetaxel	RCT	acd	NCT01905657
Ghandi2018 (16)	405	Pembrolizumab + platinum + pemetrexed	202	Placebo + platinum + pemetrexed	RCT	b	NCT02578680
Socinski2018 (17)	393	Atezolizumab + bevacizumab + carboplatin + paclitaxel	394	bevacizumab + carboplatin + paclitaxel	RCT	ac	NCT02366143
Rittmeyer2018 (18)	609	Atezolizumab	578	Docetaxel	RCT	abcd	NCT02008227
Antonia2018 (19)	475	Durvalumab maintenance	234	Placebo	RCT	abd	NCT02125461
Mok2019 (20)	636	Pembrolizumab	615	Platinum-based chemo	RCT	ac	NCT02220894
West2019 (21)	473	Atezolizumab + carboplatin + nab-paclitaxel	232	carboplatin + nab-paclitaxel	RCT	abcd	NCT02367781
Paz-Ares2019 (22)	265	Durvalumab + platinum + etoposide	266	platinum + etoposide	RCT	a	NCT03043872
Rizvi2020 (23)	740	Durvalumab + Durvalumab +Tremelimumab	352	Chemo	RCT	b	NCT02453282
Rudin2020 (24)	227	Pembrolizumab + platinum + etoposide	225	Placebo + platinum + etoposid	RCT	a	NCT03066778

The abbreviations for outcome indicators are as follows (a) The Arthralgia (b) Back pain(c) Myalgia(c) Muscular pain.

### 3.3 Risk of bias

**Figure 2:**
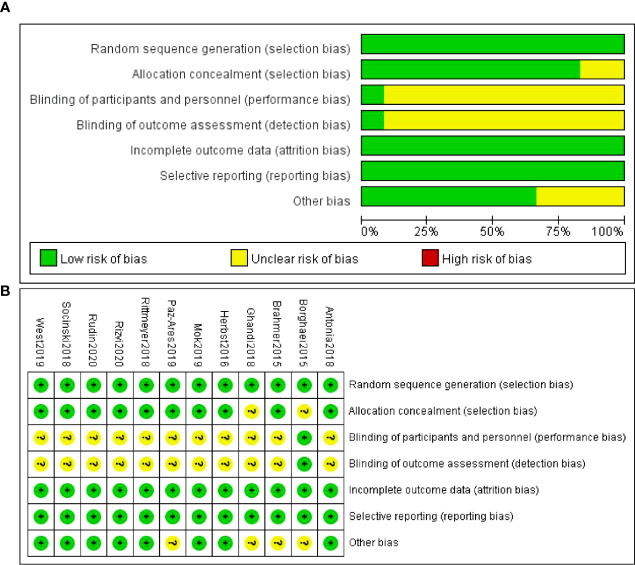
Quality assessment of the included studies. **(A)** Risk of bias summary **(B)** Risk of bias graph.

All inclueded are low risk of random sequence generation bias. Two studies are unclear about allocation concealment. Most studies are unclear of performance bias and detection bias except one studies mentioned the use of blinding methods. All studies are low risk of attritions bias and reporting bias. Some studies are unclear of other bias such are lost of follow-up (**Figure 2**).

### 3.4 Arthralgia

**Figure 3:**
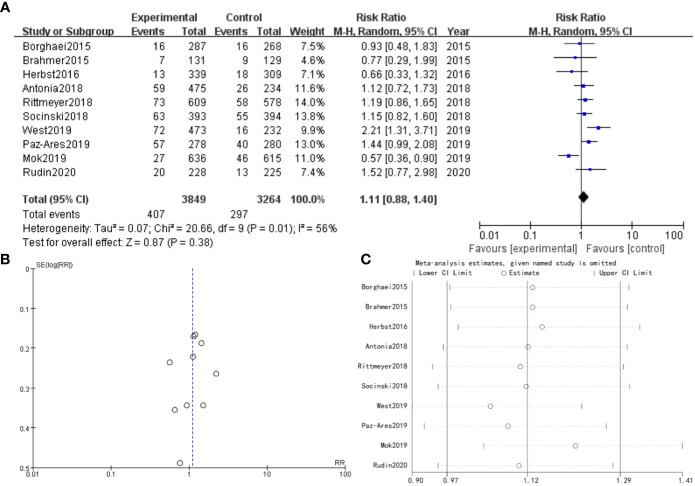
Arthralgia **(A)** Forest plot **(B)** Funnel plot **(C)** Sensitivity analysis.

There were 10 studies which discussed arthralgia. The forest plot risk ratio RR = 1.11, 95% CI [0.88, 1.40], I^2 ^= 56%. The asymmetrical shape of the funnel plot suggested the possibility of publication bias. To address this concern, a sensitivity analysis was performed and revealed that all values included in the literature fell within a reasonable range (**Figure 3**).

### 3.5 Back pain

**Figure 4:**
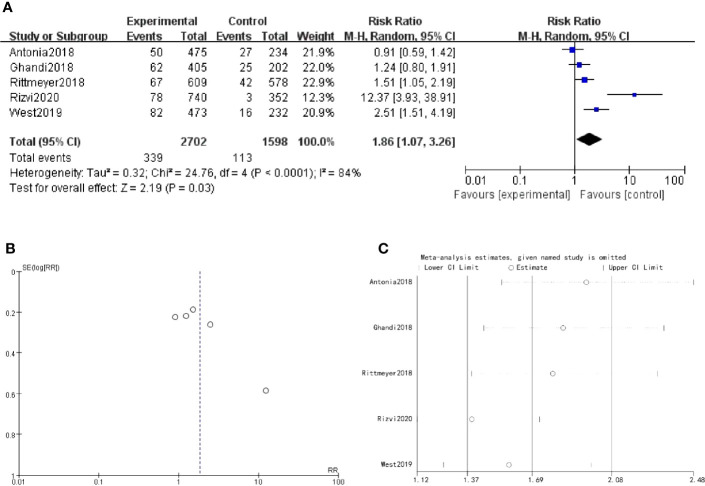
Back pain **(A)** Forest plot **(B)** Funnel plot **(C)** Sensitivity analysis.

There were 5 studies which discussed back pain. The forest plot risk ratio RR = 1.86, 95% CI [1.07, 3.26], I^2 ^= 84%. The asymmetrical shape of the funnel plot suggested the possibility of publication bias. To address this concern, a sensitivity analysis was performed and revealed that all values included in the literature fell within a reasonable range (**Figure 4**).

### 3.6 Myalgia

**Figure 5:**
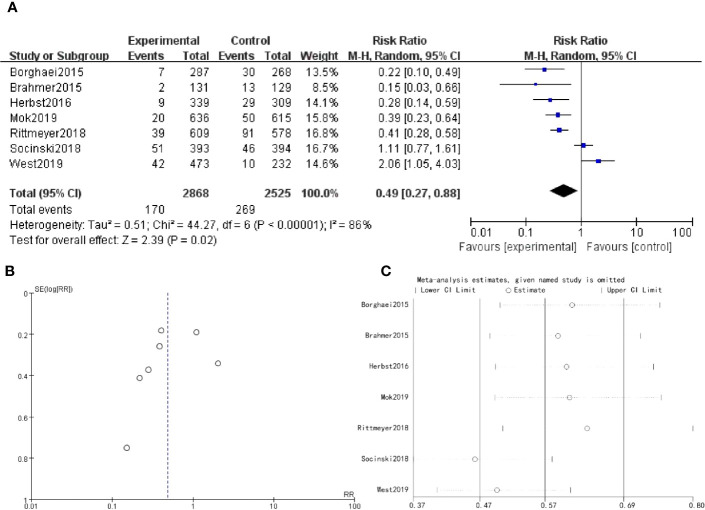
Myalgia **(A)** Forest plot **(B)** Funnel plot **(C)** Sensitivity analysis.

There were 7 studies which discussed myalgia. The forest plot risk ratio RR = 0.49, 95% CI [0.27, 0.88], I^2 ^= 86%. The asymmetrical shape of the funnel plot suggested the possibility of publication bias. To address this concern, a sensitivity analysis was performed and revealed that all values included in the literature fell within a reasonable range (**Figure 5**).

### 3.7 Muscular pain

**Figure 6:**
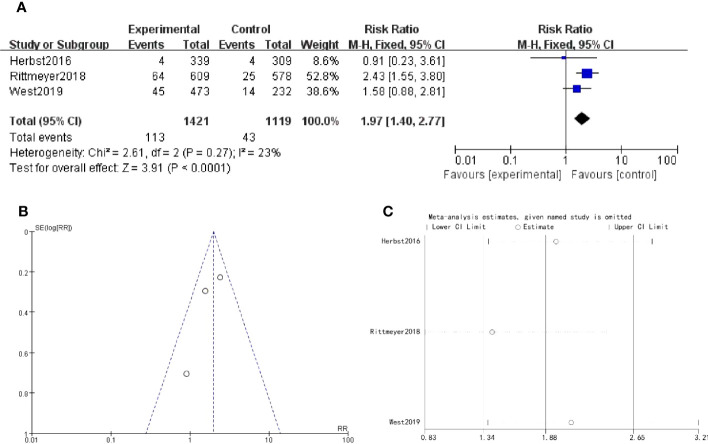
Muscular pain **(A)** Forest plot **(B)** Funnel plot **(C)** Sensitivity analysis.

There were 3 studies which discussed muscular pain. The forest plot risk ratio RR = 1.97, 95% CI [1.40, 2.77], I^2 ^= 23%. The asymmetrical shape of the funnel plot suggested the possibility of publication bias. To address this concern, a sensitivity analysis was performed and revealed that all values included in the literature fell within a reasonable range (**Figure 6**).

## 4 Discussion

Immune agents targeting Programmed cell death -1 (PD-1) are a new class of cancer drugs, which have shown excellent efficacy in the treatment of a variety of malignant tumors (25). Although these drugs have achieved great success in anti-tumor immunotherapy, their activation of the immune system may also lead to a series of autoimmune reactions, including arthralgia. However, they may also cause some side effects including arthralgia (26).

Arthralgia is a common adverse reaction. Clinical studies have shown that about 5% to 10% of cancer patients receiving targeted PD-1 immunotherapy will have arthralgia symptoms (27). This arthralgia is usually mild to moderate and can affect multiple joints, including hands, knees, shoulders, etc. It may lead to limited mobility, discomfort, and pain. The exact mechanism of arthralgia caused by targeted PD-1 immune agents is still unclear, but several hypotheses can explain this phenomenon.

Firstly, the activation of the immune system may lead to autoimmune reactions, triggering inflammatory reactions in joint tissue (28). Secondly, drugs may interfere with the Immune tolerance mechanism, causing the immune system to attack normal joint tissues. Finally, arthralgia may also be due to the influence of drugs on the neural network, resulting in sensory abnormalities and changes in pain signal transmission (26). These indicates the use of immune checkpoint inhibitors may bring a series of adverse events. Therefore, our research highlights the necessity to assess the adverse of immune-checkpoint inhibitors for lung cancer patients.

In our study, we conducted a comprehensive search across 8 databases, including PubMed, Medline (Ovid), Web of Science, Cochrane, Embase, Scopus, CKNI, Wang fang, VIP database, Sino Med, and Clinical Trials, until June 16th, 2023. A total of 3120 records were identified through the database searching process, with an additional 8 records found through other sources. After removing duplicates, we obtained 625 unique records. Among these, 51 articles were assessed for eligibility, resulting in the inclusion of 12 studies in the meta-analysis. All included studies were determined to have a low risk of random sequence generation bias. The meta-analysis result showed that arthralgia RR = 1.11, 95% CI [0.88, 1.40], I^2 ^= 56%, back pain RR = 1.86, 95% CI [1.07, 3.26], I^2 ^= 84%, myalgia RR = 0.49, 95% CI [0.27, 0.88], I^2 ^= 86% and muscular pain RR = 1.97, 95% CI [1.40, 2.77], I^2 ^= 23%. The findings indicate that the use of targeted inhibitors may lead to an increased incidence of back pain, while potentially reducing the occurrence of myalgia. However, immune-checkpoint inhibitors targeting programmed cell death-1 in lung cancer patients may not cause arthralgia and muscular pain.

This study holds significant implications for future clinical rehabilitation research. In order to enhance our comprehension of the arthralgia adverse events due to immune-checkpoint inhibitors targeting programmed cell death-1 in lung cancer patients. Our research findings can help lung cancer patients who are using immune-checkpoint inhibitors to prevent joint pain in advance. In addition, It is suggested that forthcoming studies incorporate extended follow-up periods and include a greater number of randomized controlled trials and mechanism research. Moreover, it was observed that numerous studies examined in our analysis either insufficiently reported or ambiguously reported crucial methodological particulars, such as randomization/allocation concealment and blinding methods. To enhance the reporting quality in future trials, we recommend adherence to the Consolidated Standards of Reporting Trials (CONSORT) statement (29).

## Data availability statement

The original contributions presented in the study are included in the article/supplementary material. Further inquiries can be directed to the corresponding authors.

## Author contributions

DZ: Writing – original draft. XPW: Data curation, Writing – original draft. XiW: Investigation, Writing – original draft. CL: Data curation, Writing – original draft. AW: Writing – review & editing. XiaW: Writing – review & editing. YY: Writing – review & editing. YS: Data curation, Writing – original draft.
